# The impact of closed-loop intracortical stimulation on neural activity in brain-injured, anesthetized animals

**DOI:** 10.1186/s42234-022-00086-y

**Published:** 2022-02-28

**Authors:** Marta Carè, Alberto Averna, Federico Barban, Marianna Semprini, Lorenzo De Michieli, Randolph J. Nudo, David J. Guggenmos, Michela Chiappalone

**Affiliations:** 1 Rehab Technologies, Istituto Italiano di Tecnologia, 16163 Genoa, Italy; 2 Department of Informatics, Bioengineering, Robotics System Engineering (DIBRIS), University of Genova, 16145, Genoa, Italy; 3 Aldo Ravelli Research Center for Neurotechnology and Experimental Neurotherapeutics, Department of Health Sciences, University of Milan, 20142 Milan, Italy; 4 Department of Rehabilitation Medicine, University of Kansas Medical Center, Kansas City, 66160 USA; 5 Landon Center on Aging, University of Kansas Medical Center, Kansas, 66160 USA

**Keywords:** Activity-dependent stimulation, Firing, In vivo, Micro-electrode arrays, Spike, Stroke, Synchronization

## Abstract

**Background:**

Acquired brain injuries, such as stroke, are a major cause of long-term disability worldwide. Intracortical microstimulation (ICMS) can be used successfully to assist in guiding appropriate connections to restore lost sensorimotor integration. Activity-Dependent Stimulation (ADS) is a specific type of closed-loop ICMS that aims at coupling the activity of two different brain regions by stimulating one in response to activity in the other. Recently, ADS was used to effectively promote behavioral recovery in rodent models following a unilateral traumatic brain injury in the primary motor cortex. While behavioral benefits have been described, the neurophysiological changes in spared areas in response to this type of stimulation have not been fully characterized. Here we explored how single-unit spiking activity is impacted by a focal ischemic lesion and, subsequently, by an ADS treatment.

**Methods:**

Intracortical microelectrode arrays were implanted in the ipsilesional rostral forelimb area (RFA) to record spike activity and to trigger intracortical microstimulation in the primary somatosensory area (S1) of anaesthetized Long Evans rats. An ischemic injury was induced in the caudal forelimb area through microinjections of Endothelin-1. Activity from both RFA and S1 was recorded and analyzed off-line by evaluating possible changes, either induced by the lesion in the Control group or by stimulation in the ADS group.

**Results:**

We found that the ischemic lesion in the motor area led to an overall increase in spike activity within RFA and a decrease in S1 with respect to the baseline condition. Subsequent treatment with ADS increased the firing rate in both RFA and S1. Post-stimulation spiking activity was significantly higher compared to pre-stimulation activity in the ADS animals versus non-stimulated controls. Moreover, stimulation promoted the generation of highly synchronized bursting patterns in both RFA and S1 only in the ADS group.

**Conclusions:**

This study describes the impact on single-unit activity in ipsilesional areas immediately following a cortical infarct and demonstrates that application of ADS is effective in altering this activity.

## Background

Acquired brain injuries, such as stroke, are a major cause of death and long-term disability worldwide (Organization 2018). When an ischemic stroke occurs, there is a short, hours-long window in which a resolution of the occlusion, either through mechanical or chemical interventions such as thrombectomy or tissue plasminogen activator, is possible (Phipps and Cronin 2020). Reperfusion of brain tissue is critical because, once the neurons are lost, functional and behavioral impairment will occur. If an ischemic injury occurs within the primary motor cortex (M1), as is common in stroke, there is an initial loss of descending information to the spinal cord, which leads to hemiparesis and other motor dysfunctions. In addition, there is a widespread disruption in communication throughout the sensorimotor regions such as the primary somatosensory cortex (S1) and premotor areas that is thought to contribute to the severity of the injury. While there have been investigations into the neural response to ischemic conditions at the site of injury, there is far less information on the immediate neural response of these connected regions in the period immediately following the injury.

Since only a minority of stroke survivors are able to achieve functional independence in simple activities of daily living, promoting the recovery of disabled patients is a primary challenge in scientific and clinical research (Semprini, Laffranchi et al. 2018). The standard-of-care for recovering lost functions following injury is physical therapy, which utilizes neuroplastic mechanisms to promote reorganization of spared regions and ultimately improvements in motor outcomes, but its effects are often limited or incomplete (Dimyan and Cohen 2011, Mang, Campbell et al. 2013, Belagaje 2017, Lang, Waddell et al. 2021). It is clear that there is a window of time after the initial injury in which the brain is more amenable to these neuroplastic mechanisms, starting shortly after ischemia occurs and ebbing at around 3 months (Murphy and Corbett 2009, Belagaje 2017, Coleman, Moudgal et al. 2017). Given the loss of the tissue in the ischemic core, it is likely that novel treatments will focus on the restoration of function through the spared, formerly connected areas to these regions.

To this end, we developed a treatment that reconnects brain regions that have become disconnected as a result of an acquired brain injury. Our strategy, activity-dependent stimulation (ADS) utilizes the principles of Hebbian plasticity to strengthen connections between neurons through the repetitive, reinforced synchronization of neural activity. ADS uses an online system to detect single-unit action potentials in one region to subsequently evoke activity in a different region using intracortical microstimulation (ICMS) pulses. In a model of traumatic brain injury to the caudal forelimb area in the rat (an M1 analogue), ADS was used to reconnect a premotor (rostral forelimb area, RFA) area with primary somatosensory cortex (S1). This treatment was started within several hours of injury and was applied continuously for three weeks, but behavioral improvements were evident within one week and were restored within two. Further, we observed a shift in the neural activity within RFA that was not present with random stimulation (Guggenmos (2013)). To further characterize ADS, we have investigated its ability to rapidly alter firing characteristics in both anesthetized and ambulatory rats without brain injury (Averna, Pasquale et al. 2020, Averna, Hayley et al. 2021). However, due to the nature of cortical injury and the subsequent global disruption in activity and communication, it was unknown what impact ADS would have on the ability to facilitate changes in activity within the trigger and target regions. The purpose of this study is to evaluate *i)* the global impact of a focal lesion on the neuronal activity of spared premotor and somatosensory areas and *ii)* the ability of acute ADS to significantly alter the lesion induced changes. We performed acute experiments in anesthetized rats using a model of focal ischemic lesion in the primary motor area (caudal forelimb area, CFA) using micro-injections of a potent vasoconstrictor, i.e. Endothelin-1 (ET-1) (Frost, Barbay et al. 2006). Neurophysiological activity of both the rostral forelimb area (RFA) and the somatosensory cortex (S1) was recorded by using micro-electrode arrays. ADS was utilized following the same procedures as previous studies (Averna, Pasquale et al. 2020, Averna, Hayley et al. 2021), by pairing the occurrence of the single-unit activity on a selected channel within RFA with ICMS applied to S1 after CFA ischemic injury.

Understanding the shifts in the neural response following brain injury and coupling this response to our closed-loop stimulation paradigms offers a novel way to probe cortico-cortical circuits and has the potential to inform development of future therapies for acquired brain injury.

## Methods

### Animals

**Fig. 1:**
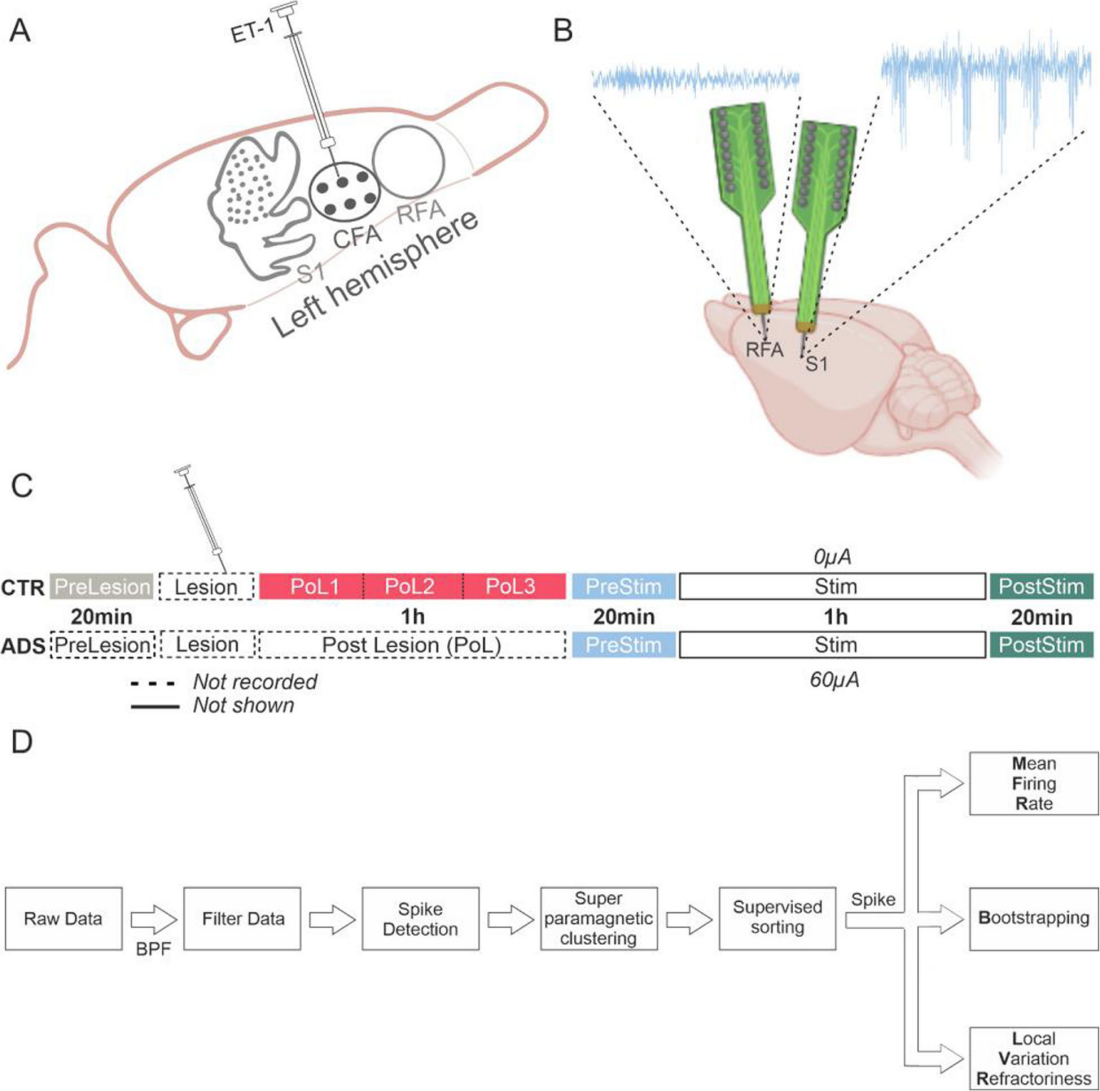
Experimental protocol. **A)** Areas of interest were stereotaxically located under anesthetized conditions. Two multisite MEAs (see **B** for details) were placed in the left hemisphere, i.e. in the Rostral Forelimb Area – RFA for recording, and in the Primary Somatosensory area - S1 for recording and stimulation. **B)** Schematic representation of the setup (created with BioRender.com). Two 4 shank, 16 contact site MEAs were inserted into the cortex. Top: raw signals of spontaneous activity during the PreStim phase (see **C** for details) in the recording sites (i.e. RFA and S1). **C)** Experimental timeline of the two experimental groups, i.e. CTR (Control – lesioned, not stimulated) and ADS (lesioned and stimulated). Both CTR and ADS were characterized by one phase of lesion induction, where no recording was performed (dotted line). Control experiments (top sketch) were characterized by five phases of recording spontaneous activity (i.e., PreLesion - grey, PreStim - light blue and PostStim - green of 20 min each, PostLesion and Stim of 60 min each). For the Stim phase, the stimulation current was set to 0A for the entire recording time of 60 min. During data analysis PostLesion was split into three 20 min subphases (i.e., PoL1, PoL2, and PoL3 - cherry red). The ADS experiments (bottom sketch) were characterized by three phases, the middle one consisting of 60 min of stimulation (Stim) where current was delivered in the form of biphasic squared pulses at 60 μA, while the other two were characterized by 20 min of spontaneous activity recording, during which no stimulation was applied (i.e., PreStim and PostStim). **D)** Block diagram of data analysis. Briefly, raw data was filtered with a bandpass filter (300–3000 Hz); a custom offline spike detection was used to perform spike discrimination; a superparamagnetic clustering sorted the detected spikes and finally a supervised visual assessment of sorted clusters was used to validate the spike profiles. Spike profiles were then utilized for Mean Firing Rate, Bootstrapping and Local Variation compensate for Refractoriness (LvR) analyses (see text)

Nine adult, male Long-Evans rats (weight: 350-400 g, age: 4–5 months; Charles River Laboratories, Wilmington, MA, USA and Charles River Laboratories Italia SRL, Calco, Italy) were utilized in this study. Rats were divided into either the lesioned, no stimulation group (i.e. Control, CTR; *n* = 3) or the lesioned, stimulation (i.e. Activity-Dependent Stimulation, ADS; *n* = 6) group (Fig. 1). The experiments were performed both in the USA and in Italy. The University of Kansas Medical Center Institutional Animal Care and Use Committee (USA: protocol 2017–2384 approved on 2/17/17) and the Italian Ministry of Health and Animal Care (Italy: authorization ID 861/2015 PR) approved all experiments performed for this study.

### Surgical procedures

#### Preparation

Rats were assigned to one of the two groups prior to the surgical procedure. Anesthesia was initially performed by placing the rat inside a chamber attached to a vaporizer and introducing gaseous isoflurane (5% @ 1 lpm) until induction. A surgical level of anesthesia was obtained by administering ketamine (80–100 mg/kg IP) and xylazine (5–10 mg/kg) and was maintained throughout the entire procedure by bolus injections of ketamine (10–100 mg/kg/hr. IM) as needed, whenever a positive pinch or ocular reflex was detected. After securing the rat in the stereotaxic frame, it was placed on a homeostatically controlled heating blanket; temperature and vital parameters were monitored over the duration of the procedure. Lidocaine cream was applied as a topical analgesic prior to performing a midline skin incision spanning rostro-caudally between ~ 6 mm rostral to bregma and ~ 5 mm distal to the atlanto-occipital junction exposing the skull surface. The muscles of the neck overlying the Cisterna Magna were reflected and a laminectomy was performed in the spinal dura to allow cerebrospinal fluid (CSF) to drain, mitigating brain edema during and after the craniotomy. As shown in Fig. 1A, six 0.65-mm diameter holes were drilled into the skull over the left hemisphere (contralateral to the preferred forelimb) in the area corresponding to the caudal forelimb area (CFA; the M1 forelimb representation analogue in rats) as follows: + 0.5,+ 2.5; 1.5,+ 2.5; 2.5, + 2.5; + 0.5,+ 3.5; 1.5,+ 3.5; 2.5, + 3.5AP,ML from bregma. Based on standard stereotaxic locations (Kleim, Bruneau et al. 2003), burr holes (3 mm diameter) over RFA and S1 were made at + 3.5, + 2.5 and − 1.25, + 4.25 AP, ML, respectively. Once the skull was exposed, the dura mater was removed in these burr holes (RFA and S1) to allow insertion of microelectrode arrays (MEAs; A4x4-5 mm-100-125-703-A16, NeuroNexus; Fig. 1B, schematic representation).

#### Lesion

An ischemic injury was induced in CFA by intraparenchymal injection of Endothelin-1 (ET-1, Bachem Americas, USA), a potent vasoconstrictor: 0.33 μl ET-1 was injected at a rate of 3 nl/sec via a 160 μl pipette (o.d.) attached to a 1 μl Hamilton syringe ~ 1.5 mm below the pial surface in each of the 6 holes over CFA (Gilmour (2005)). This procedure produced an infarct that encompassed CFA, confined to an area of 0.5 mm diameter, while leaving RFA and S1 intact (Fang (2010)).

### Recording/stimulation paradigms

Locations for electrode insertions were based on stereotaxic coordinates and blood vasculature patterns. As depicted in Fig. 1B, RFA was implanted with a four-shank, sixteen-contact site electrode with 1–1.5 MΩ impedance at each site (A4x4-5 mm-100-125-703-A16, NeuroNexus) at a depth of 1700 μm. In S1, a similar four-shank, sixteen-contact site electrode was inserted in S1 at a depth of 1200 μm. The S1 electrode had an activated electrode site within the array, dropping the impedance of this site to roughly 0.2 MΩ (contact 6, activated A4x4-5 mm-100-703-A16, NeuroNexus) to allow for stimulation. Electrodes were activated by the manufacturer following the procedure described in https://www.neuronexus.com/files/technicalsupportdocuments/Activation.pdf, and verified by the investigators with impedance testing through the Intan RHS system. Continuous extracellular signals from all channels were amplified, digitized and stored at a sampling rate of 30 kHz using standard, commercially available neurophysiological hardware (Intan RHS, Intan technologies LLC).

For stimulation procedures, a single channel in RFA was selected based on visual observation of spike amplitudes and signal to noise ratios. Once a unit was identified, a channel-specific threshold was set in the Intan software to act as the trigger for activity-dependent stimulation (ADS). Upon each threshold crossing, a single stimulation pulse was delivered to channel 6 on the S1 probe. To prohibit feedback from stimulus-evoked RFA spikes and stimulus artifacts from triggering stimulation, a blanking period (28 ms) followed each stimulus limiting the maximum stimulation rate to roughly 35 Hz. Each triggered stimulus pulse was a single charged-balanced biphasic, cathodal-leading pulse (200 μs positive, 200 μs negative). In the control animals, the pulse current was set at 0 μA and for ADS animals this current was set at 60 μA. All ground pins (for amplification and stimulation) were required to be kept at the same potential. Therefore, all ground pins were bridged and tied to a common point on the animal, either to a skull screw in the intraparietal bone or to a needle embedded in the clavotrapezius muscle.

### Experimental protocol

Experiments were carried out at both the University of Kansas Medical Center (US) and the Istituto Italiano di Tecnologia (Italy) by the same investigators. Due to differences in stereotaxic frames, attachments, and holders, the methodology was modified at the respective locations and was updated as a result. In both settings, there was a difference of roughly 5–10 min between the insertion of one probe and the insertion of the second probe. Probes were attached to the neurophysiological equipment prior to insertion. Upon insertion, signals were monitored to ensure that single-unit activity could be detected; impedance measurements were taken and parameters were determined before data recording began. As described in the ‘Animals’ section, rats were assigned to one of the two groups: CTR (*n* = 3) and ADS (*n* = 6) which had slightly different experimental protocols as detailed in Fig. 1C. In the CTR group, electrodes were placed in the cortex prior to the lesion induction allowing a period of pre-lesion (i.e., PreLesion - PreL, grey) and post-lesion (i.e., PostLesion - PoL, cherry red) recording phases. In the ADS group, due to technical limitations, the electrodes had to be placed after the induction of the ischemic lesion, limiting pre-lesion data to the CTR group (Fig. 1 C, dashed line). In both groups, there was a common 100-min period of recording beginning one hour after the final ET-1 injection which consisted of a 20-min pre-stimulation period (i.e., PreStim - PreS, light blue in Fig. 1 C), a 60-min stimulation period (i.e., Stim, white in Fig. 1 C) and a 20-min post-stimulation period (i.e., PostStim - PoS, green in Fig. 1 C).

### Data processing

All data from the two groups was processed in the same way. Data was initially filtered using a 4th order elliptic bandpass filter in the range of 300–3000 Hz to remove low frequency components within the signal. A custom semi-automatic spike discrimination approach was used to detect and sort spikes from the filtered data. This consisted of a custom offline spike detection algorithm, called Precise Timing Spike Detection (PTSD). PTSD has several parameters that are user-configurable. Peak Lifetime Period (i.e., PLP, sized to contain at most one single spike, set at 2 ms) and the Relative Maximum/Minimum, a peak-to-peak amplitude differential threshold, based on the standard deviation of the noise. This spike detection algorithm ensures that candidate spikes have appropriate parameters to isolate individual units (more details in Maccione, Gandolfo et al. 2009). Spike detection was followed by the freely available superparamagnetic clustering technique developed by the Quiroga group (Quiroga, Nadasdy et al. 2004, Mohammed H 2016) Using a custom Matlab pipeline, spike profiles were validated through a supervised visual assessment of sorted clusters by a single investigator for the entire dataset. This stage is akin to manual “cluster-cutting”, in which the user has a visual indicator of selectable spike waveform characteristics (typically the peak-to-peak amplitude). The time frame considered for a spike extends 0.4- ms prior to 0.8-ms after each detected peak (refer to the Matlab code at: https://github.com/m053m716/CPLtools/tree/master/MatlabAddons/Functions/Spike%20Analyses).

Peaks that clearly corresponded to noise were excluded at this stage by assignment to a “noise” or “non-neural” source cluster. All subsequent analyses were performed only on the units identified as neural spikes during the sorting process. For the CTR group, to study the effects of a short-term lesion, we split the 1-h recording session of PostLesion (PoL) into three sequential 20-min sub phases (i.e. PoL1, PoL2 and PoL3 as reported in Fig. 1C).

#### Mean firing rate

We evaluated the level of neuronal firing by computing the mean firing rate (MFR, spikes/s) in each experimental phase. We set a minimum firing rate of 0.01 spikes/s of all prospective units as the threshold for consideration for analysis (Averna, Hayley et al. 2021).

#### Bootstrapping method

For each recording phase of the two groups (i.e., CTR and ADS), we quantified a significant deviation from a null (zero centered) distribution of the differences in firing rates between two time-points for a given unit. Using a bootstrapping method (Slomowitz E 2015, Averna, Pasquale et al. 2020) two time-segments were divided into 1-min bins and then randomly shuffled 10′000 times into two groups. Subsequently, a null-distribution was produced by differences between the means of the two randomly shuffled groups. The real difference falling outside the 95% confidence interval of the zero distribution was considered significant.

#### Local variation compensate for refractoriness

A revised version of the Lv parameter, called Local Variation compensate for Refractoriness (LvR) as proposed in (Shinomoto, Kim et al. 2009), was used to describe the intrinsic firing irregularity of singular neurons. LvR evaluates the local variation of the ISI, assuming that rate dependence is induced by the refractory period of a spike, R, which is subtracted from the interspike interval (ISI). The refractoriness constant, R, was set to 5 ms to intensify the characterization of firing dynamics of individual neurons in terms of F values (Shinomoto, Kim et al. 2009). The final revised local variation LvR equation is defined as:

$$ LvR=\frac{3}{n-1}\sum \limits_{i=1}^{n-1}\left(1-\frac{4{I}_i{I}_{i+1}}{{\left({I}_i+{I}_{i+1}\right)}^2}\right)\left(1+\frac{4\mathrm{R}}{\left({I}_i+{I}_{i+1}\right)}\right) $$

Where *Ii* and *Ii + 1* are the *i*-th and *i*+1st ISIs and *n* is the number of ISIs. The value provided by this metric, ranged from 0 to more than 2, is useful to classify the individual neuron’s activity into Regular (approx. 0.5 ± .25), Random (approx. 1 ± 0.25) and Bursty (approx. 1.5 ± 0.25) firing patterns (Shinomoto, Kim et al. 2009).

### Statistical analysis

Statistical analysis was performed in Matlab (The MathWorks, Natick, MA, USA). Data was first tested for normality with the Kolmogorov-Smirnov test. As our data failed the normality test, we used non-parametric tests for our analysis. We performed the Friedman test to detect differences among firing rates at different experimental phases and used the Tukey-Kramer test for post-hoc analysis. We used the Wilcoxon signed-rank test to assess statistical differences pre- and post-stimulation within experimental groups, and the Wilcoxon rank-sum test to assess statistical differences among different experimental groups. *P*-values < 0.05 were considered as significant and corrected for multiple comparisons when relevant.

## Results

### An ischemic lesion induced changes of firing activity

**Fig. 2:**
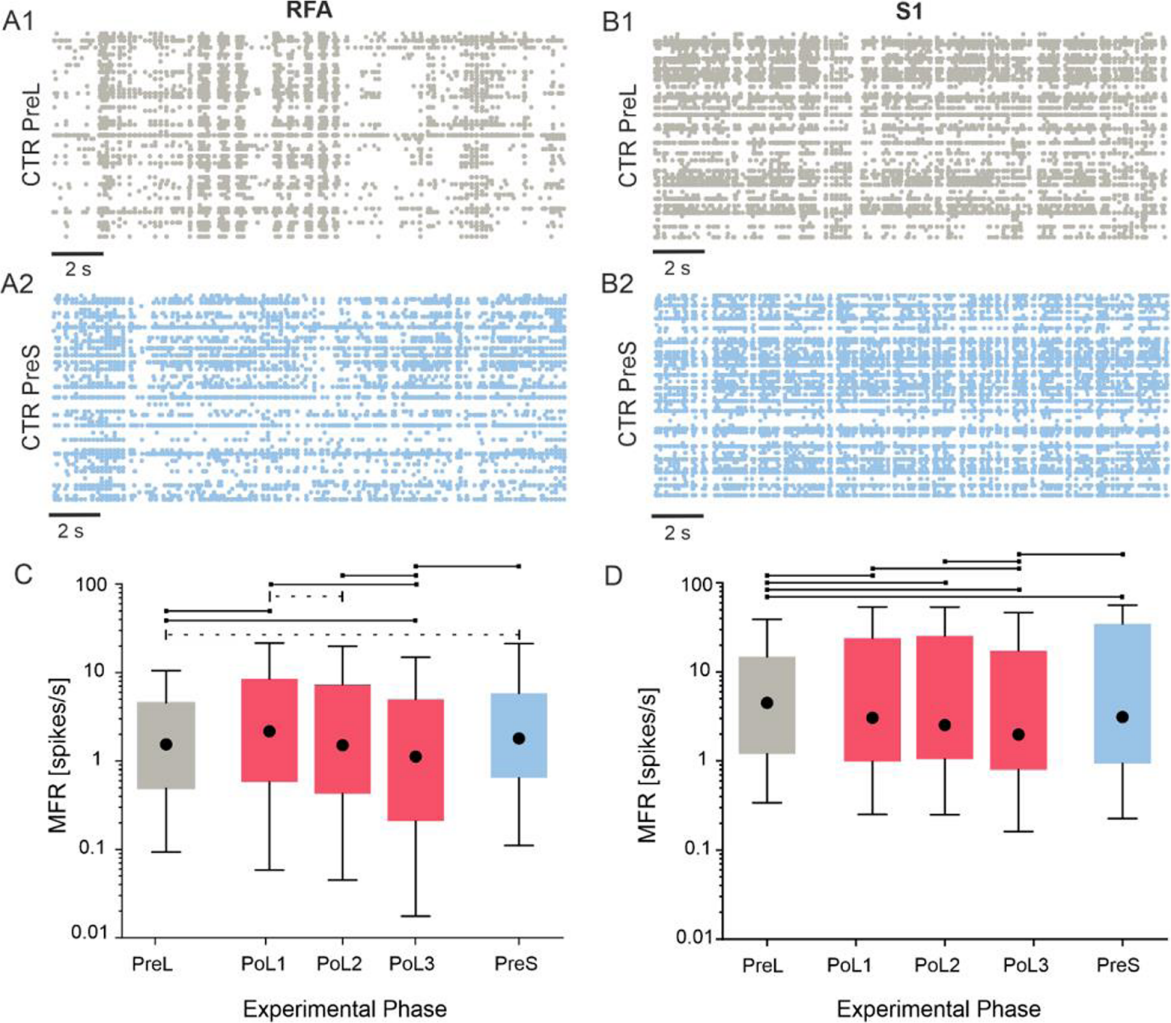
Effect of the lesion on firing rate. **A)** 20-s raster plots of the activity recorded in RFA in a representative Control (CTR) experiment: Pre Lesion - PreL phase (**A1**), Pre Stim phase - PreS (**A2**). **B)** 20-s raster plots of the activity recorded in S1 in a representative Control (CTR) experiment: Pre Lesion - PreL phase (**B1**), Pre Stim phase - PreS (**B2**). Each single dot represents a spike; each line of the raster plot reports the activity of a single unit. **C)** Box plot of the Mean Firing Rate (MFR, spikes/s) of RFA for the entire dataset of CTR experiments. **D)** Box plot of the Mean Firing Rate (MFR, spikes/s) of S1 for the entire dataset of CTR experiments. For each box plot (**C-D**), the central black dot indicates the median, the box limits indicate the 25th and 75th percentiles. Whiskers represent the 5th and the 95th percentiles. Dashed line p < 5·10^− 2^; solid line *p* < 5·10^− 5^ Friedman non parametric test. Color code as in Fig. 1C

We evaluated the mean firing rate of RFA and S1 in the CTR group before and after the ischemic lesion. Specifically, we evaluated how the spiking activity of all identified units was impacted by the lesion and how this evolved over time in 20 min increments (Fig. 2). Baseline (Pre Lesion, PreL) activity was characterized by a mix of random spiking and sparse synchronous events across the different electrodes within an MEA in both RFA and S1, as shown in representative raster plots of one rat (Fig. 2A). After the lesion (i.e., Pre Stim, PreS, about 1 h after the lesion induction), there was a shift in activity from pre-lesion levels (Fig. 2B). In both the PreL and PreS periods, activity in S1 was characterized by a higher level of firing than in RFA (4.49 spikes/s in S1 vs. 1.54 spikes/s in RFA PreL; 3.11 spikes/s in S1 vs. 1.8 spikes/s in RFA PreS).

In RFA (Fig. 2C), the lesion induced an overall significant increase in the MFR from the PreL to the PreS period (1.54 spikes/s PreL vs:1.8 spikes/s PreS *p < 5·10^− 2^ Friedman nonparametric test). Significant increases were also observed from the PreL period to PoL1 and from PoL3 to PreS periods (1.54 spikes/s - PreL vs: 2.15 spikes/s - PoL1 **p < 5·10^− 5^; 1.12 spikes/s PoL3 vs 1.8 spikes/s PreS, respectively. **p < 5·10^− 5^ Friedman nonparametric test). Significant decreases in MFR were observed from PreL to PoL3 (1.54 spikes/s - PreL vs 1.12 spikes/s PoL3), from PoL1 to PoL2 and PoL3 (2.15 spikes/s PoL1 vs: 1.5 spikes/s PoL2 *p < 5·10^− 2^; 2.15 spikes/s PoL1 vs 1.12 spikes/s PoL3) and from PoL2 to PoL3 (1.5 spikes/s PoL2 vs 1.12 spikes/s PoL3).

S1 exhibited the opposite trend (Fig. 2D), characterized by a significant decrease in the MFR from the PreL to the PreS period (4.49 spikes/s PreL vs 3.11 spikes/s PreS, **p < 5·10^− 5^ Friedman non parametric test). This decreasing trend was observed for all PoL periods with respect to PreL (4.49 spikes/s PreL vs 3.04 spikes/s PoL1, 2.52 spikes/s PoL2, 1.97 spikes/s PoL3, **p < 5·10^− 5^ Friedman non parametric test) and from PoL1 and PoL2 to PoL3 (3.04 spikes/s PoL1, 2.52 spikes/s PoL2 vs: 1.97 spikes/s PoL3, **p < 5·10^− 5^ Friedman non parametric test). There was an observed recovery of firing rate between PoL3 and PreS (1.97 spikes/s PoL3 vs 3.11 spikes/s PreS, **p < 5·10^− 5^ Friedman non parametric test).

### An ischemic lesion affected the number of firing units

**Fig. 3:**
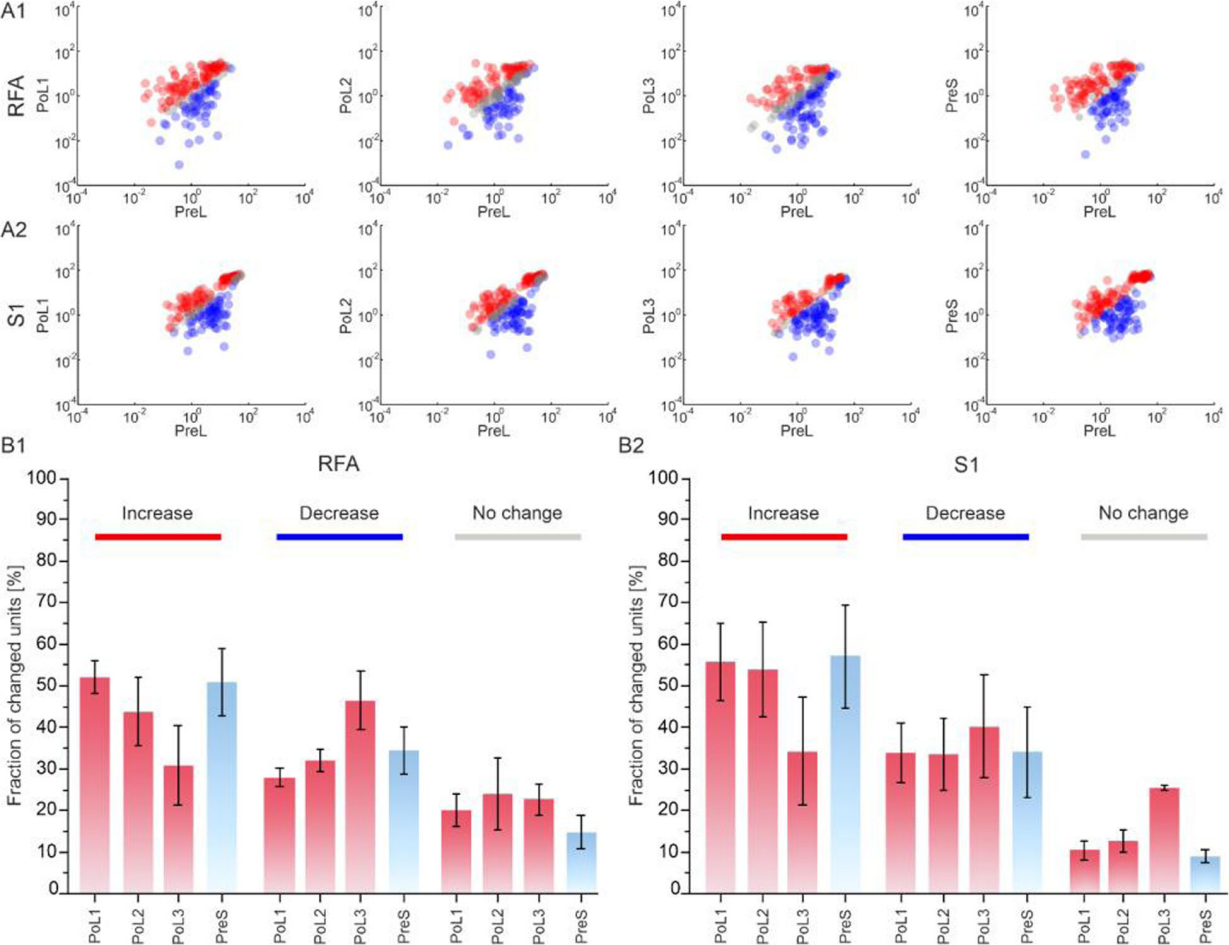
Effect of the lesion on the firing units. Per unit correlation for RFA (**A1**) and S1 (**A2**) between baseline firing rates (x-axis, PreLesion (PreL)) and firing rates of immediately post lesion phase and pre stimulation session (y-axis, PostLesion (PoL1–3), Pre Stim (PreS)), respectively. Data is shown in scatter plots, where colors represent units that significantly (i.e. according to the bootstrapping methods described in the Materials and Methods) increased (red), decreased (blue), or remained stable (grey). The global number of units in RFA (**A1**) was 54.7 ± 3.8; in S1 (**A2**) the total number of units was 58.7 ± 9.5. Bottom: **B1)** for RFA and  **B2)** for S1. Average fraction of units that increased (red bar), decreased (blue bar) or no changed (grey bar) their firing with respect to the baseline period of recording (i.e. PreLesion, PreL) calculated for the immediately post lesion phase (i.e. PostLesion, PoL1–3, red) and pre stimulation session (i.e. PreStim, PreS, light blue). Data are reported as mean ± SEM (standard error of the mean)

To compare single unit firing activity in the five experimental phases (i.e., PreL, PoL1, PoL2, PoL3 and PreS) and identify possible changes, we evaluated whether the difference in values between two considered phases (i.e., PreL vs. PoL1–3 and PreL vs PreS) for a given unit significantly deviated from a null (zero centered) distribution using a bootstrapping method (see the ‘Data Processing - Bootstrapping Method section in the Materials and Methods), as reported in Fig. 3A. We calculated the fraction of units whose firing significantly increased, decreased or remained constant (i.e., according to the bootstrapping method) between *i)* the Pre Lesion and each of the PoL1, PoL2 and PoL3 phases (Fig. 3A1, panels from left to right) in RFA, and *ii)* the Pre Lesion and each of the PoL1, PoL2 and PoL3 phases (Fig. 3A2, panels from left to right) in S1.

In case *i)*, during the immediate Post Lesion phase, about 52% of the units showed an increase in the firing rate. This percentage decreased across the entire Post Lesion condition reaching as low as 30%, returning to values around 50% during Pre Stim (Fig. 3B1). The number of units that decreased their firing with respect to the baseline constantly increased during the Post Lesion phase, up to 44%, with a decrease in the PreS, where the percentage reached values around 35%. The number of units that showed no change was stable for the entire PoL1–3 and PreS phases, around 16–25%.

In case *ii)*, during the immediate Post Lesion phase (i.e. PoL1), about 55% of the units show an increase in the firing rate. This percentage decreased during both PoL2 and, in a greater way, PoL3, reaching the minimum value of 34% (Fig. 3B2). In PreS, we observed a jump in the number of increased units (i.e. 57%), with values even higher than the Pre Lesion condition. The number of units that decreased their firing with respect to the baseline slightly increased during the Post Lesion phase but returned to values comparable to the Pre Lesion during PreS condition. As in RFA, the number of units that showed no change was stable for the entire PoL1–3 and PreS phases, with values in the range 8–25%.

### ADS increased the global level of firing

**Fig. 4:**
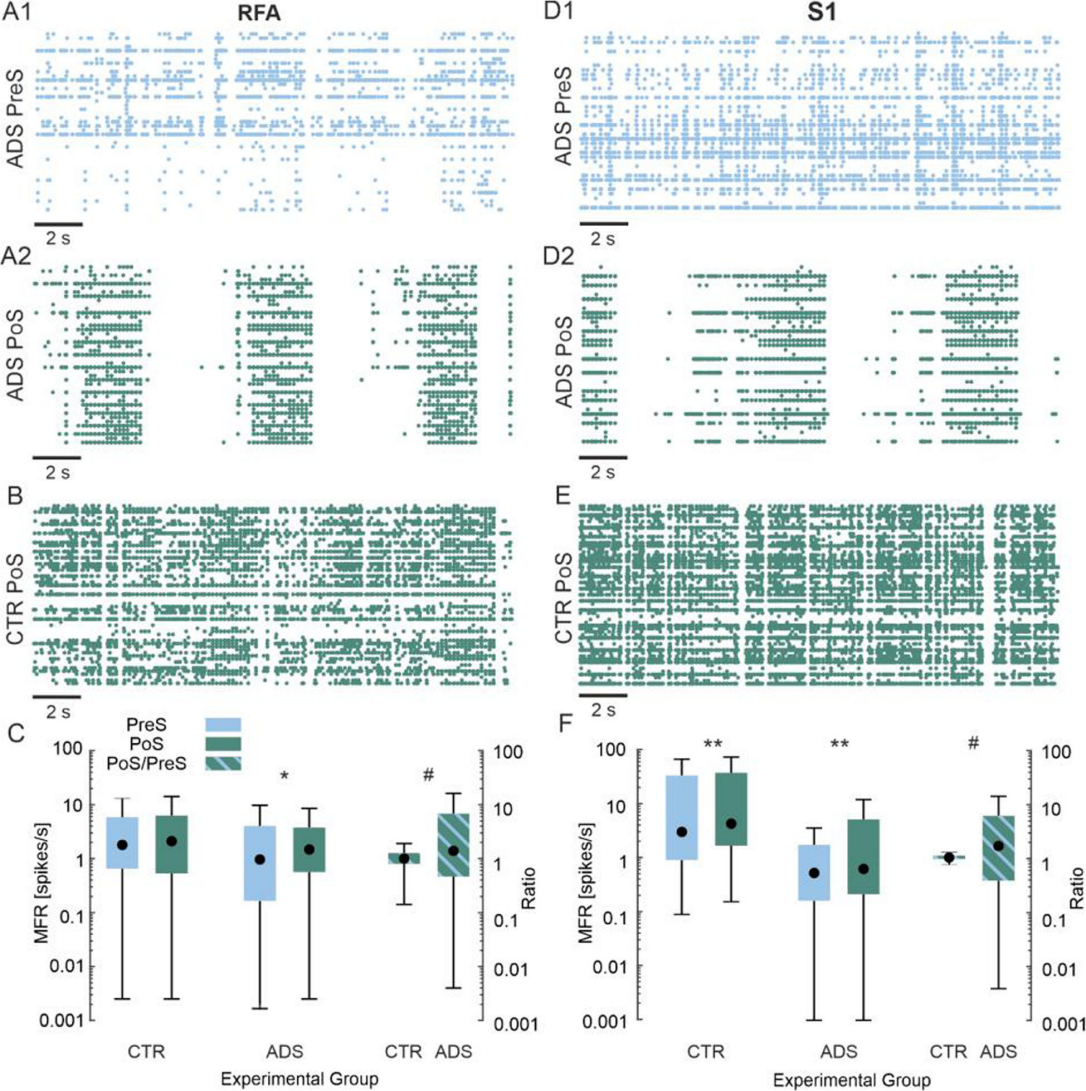
Effect of the stimulation on the firing rate: ADS vs CTR. **A)** 20-s raster plots of the activity recorded in RFA in a representative Activity-Dependent Stimulation (ADS) experiment: Pre Stim phase - PreS (**A1**) and Post Stim phase - PoS (**A2**). **B)** 20-s raster plot of the activity recorded in RFA in a representative Control (CTR) experiment: Post Stim phase - PoS (**B**). **C)** Box plot of the Mean Firing Rate (MFR, spikes/s) of RFA for the entire dataset of CTR and ADS experiments. **D)** A 20-s raster plot of the activity recorded in S1 in a representative Activity-Dependent Stimulation (ADS) experiment: Pre Stim phase - PreS (**D1**) and Post Stim phase - PoS (**D2**). **E)** A 20-s raster plot of the activity recorded in S1 in a representative Control (CTR) experiment: Post Stim phase - PoS (**E**). **F)** Box plot of the Mean Firing Rate (MFR, spikes/s) of S1 for the entire dataset of CTR and ADS experiments. For each box plot (**C- F**), the central black dot indicates the median, the box limits indicate the 25th and 75th percentiles. Whiskers represent the 5th and the 95th percentiles. *p < 8·10^− 3^, **p < 8·10^− 5^ Wilcoxon signed rank test; ^#^*p* < 8·10^− 3^ Wilcoxon rank-sum test. Color code as in Fig. 1C

We evaluated the level of firing of RFA and S1 in both the ADS and CTR group, before (i.e., Pre Stimulation, PreS) and after the stimulation (i.e. Post Stimulation, PoS) phase (Fig. 4). In the ADS group in RFA, the baseline activity PreS was characterized by random spiking with some synchronous events, as shown in the raster plot of one representative ADS experiment (Fig. 4A1). After the closed-loop stimulation, in the PoS phase, we observed a global increase of activity, with massive network-wide synchronous events (Fig. 4A2) which was not observed in the PoS of the Control group (CTR), as shown by a CTR representative raster plot (Fig. 4B), where a sustained level of firing is exhibited, but no collective, synchronous patterns are present. No difference was found in the CTR group (1.8 spikes/s PreS vs 2.1 spikes/s PoS: n.s., Wilcoxon signed rank test), while the ADS exhibited a significant increase in the level of firing after the stimulation session (0.96 spikes/s PreS vs 1.48 spikes/s PoS: *p < 8·10^− 3^, Wilcoxon signed rank test). The ratio between PoS and PreS (Fig. 4C) highlighted the significant increase induced by the stimulation in the ADS group with respect to CTR. This indicates that the closed loop stimulation was able to significantly increase the global level of firing in RFA.

In both groups in S1, a similar trend to RFA was observed. The raster plot of a representative ADS experiment is shown in Fig. 4D. Similar spiking characteristics to RFA were observed in S1 (Fig. 4D1). After the stimulation, there was also a general increase of activity characterized by network-wide bursting (Fig. 4A2). In the CTR experiment, after the 0 μA stimulation, the level of firing is higher than in ADS (Fig. 4F) but characterized by less synchronicity. The PreS and PoS conditions for both CTR and ADS had a statistically significant increase in activity both in CTR and ADS (3.11 spikes/s PreS vs 4.43 spikes/s PoS for CTR and 0.54 spikes/s PreS vs 0.64 spikes/s PoS for ADS: **p < 8·10^− 5^ Wilcoxon signed rank test).

### ADS affected the number of firing units

**Fig. 5:**
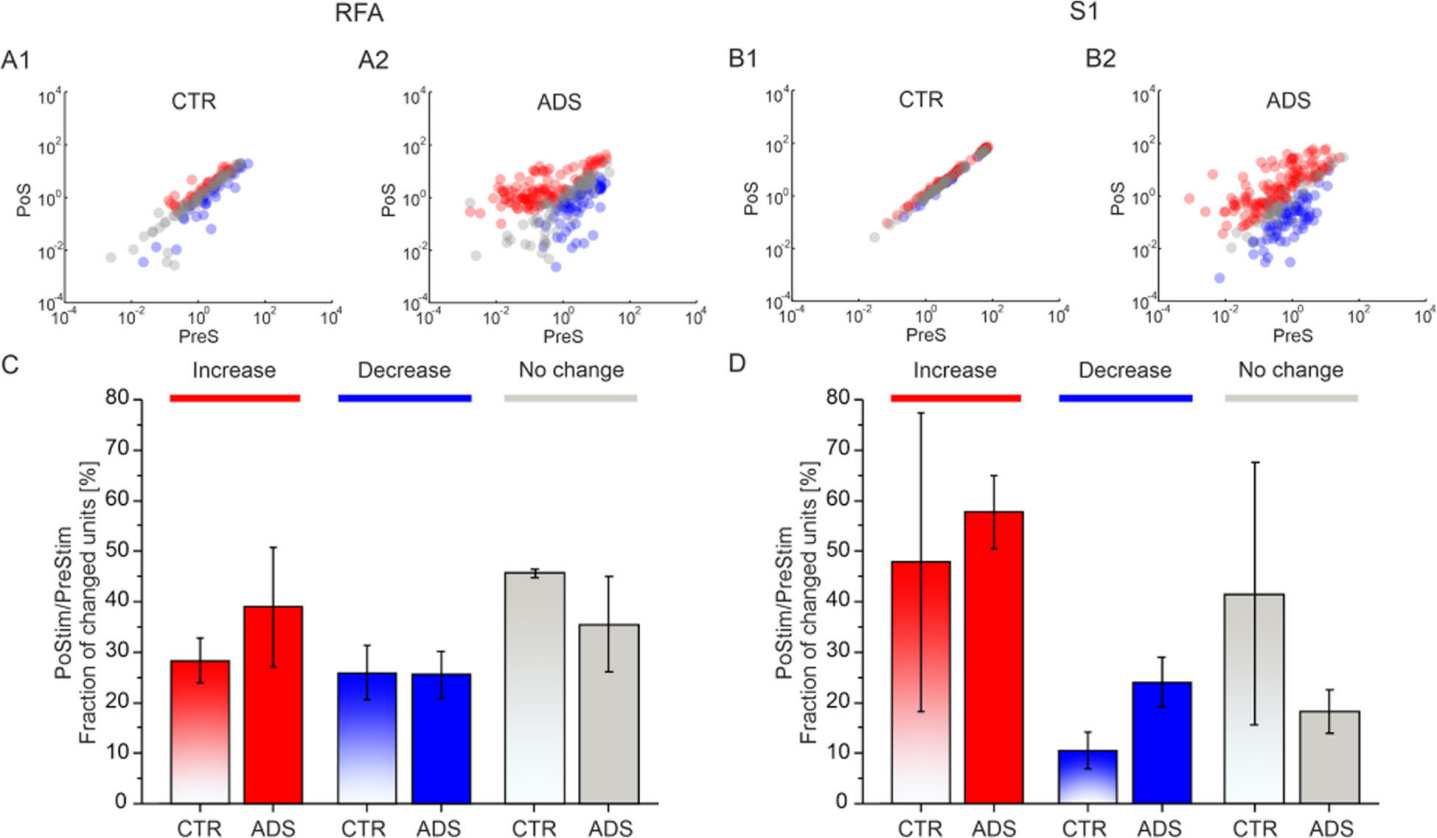
Effect of the stimulation on the firing units: ADS vs CTR. On the left, data for RFA area. In **A1)** for CTR and **A2)** for ADS, per unit correlation between baseline firing rates (x-axis, PreStim - PreS) and firing rates after the stimulation session (y-axis, PostStim - PoS). Data is shown in scatter plots where colors represent units that increased (red), decreased (blue), or remained stable (light grey). In **C)** for both CTR (shaded bars) and ADS, average fraction of units that increased (red), decreased (blue) or no changed (grey) their firing with respect to the baseline period of recording (i.e. PreStim - PreS) calculated for the immediately post stimulation phase (i.e. PostStim - PoS). Data are reported as mean ± SEM (standard error of the mean). Statistics. The same representations are on the right for S1 area. In **B1)** left sketch CTR and **B2)** right sketch ADS and in **D)** the average fraction of units that changed their firing in both CTR and ADS

To compare single unit firing activity before and after the stimulation and evaluate possible changes, the difference in values between the two phases (i.e., PoS vs PreS) for a given unit significantly deviated from a null (zero centered) distribution using a bootstrapping method (see the ‘Data Processing - Bootstrapping Method section in the Materials and Methods), as reported in Fig. 5A, B. The fraction of units whose firing significantly increased, decreased or remained constant (i.e. according to the bootstrapping method) in RFA (Fig. 5A) and in S1 (Fig. 5B) was calculated. The quantification of the previous results is reported in Fig. 5C for RFA and Fig. 5D for S1. In Fig. 5C (RFA), after the stimulation, about 28% of the units in CTR and 38% of the units in ADS show an increase in the firing rate. The percentage of decreased units in PoS vs PreS is around 25% for both CTR and ADS. The number of units that showed no change is around 45% in CTR and 35% in ADS, but with ADS exhibiting higher variability. In Fig. 5D (S1), the number of increased units is around 58% in ADS while is 48% in CTR, but highly variable. In S1, the number of decreased units is higher in ADS (24%) than in CTR (10%), while the number of no change units is high and very variable in the CTR (41%) with respect to the ADS (18%) group.

### ADS induced a shift from random firing to bursting activity

**Fig. 6:**
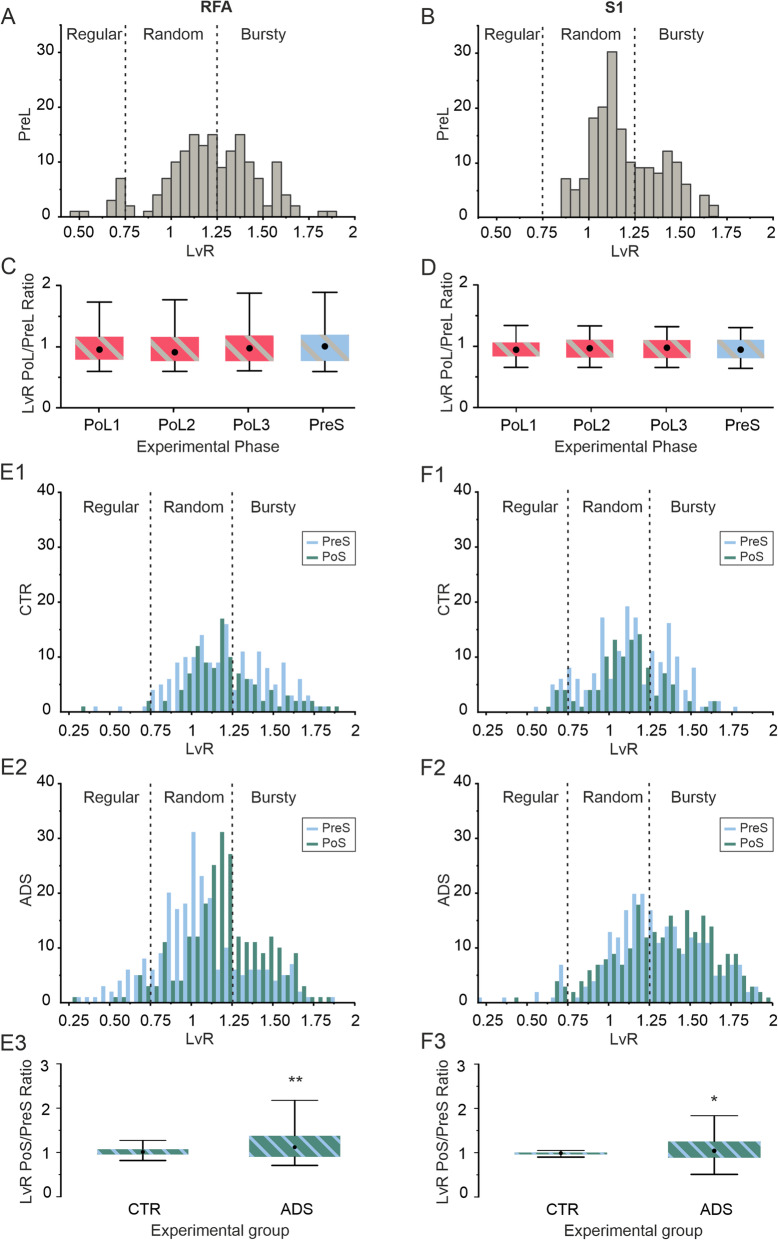
LvR analysis. Panels on the left refer to RFA area, panels on the right refer to S1 area. In **A)** LvR distribution shown as histogram with a bin size of 0.05 and determined across all the single units belonging to RFA during pre-lesion phase. In **B)** LvR distribution shown as histogram with a bin size of 0.05 and determined across all the single units belonging to S1 during pre-lesion phase. In **C)** Box plot for the CTR group of the LvR calculated between baseline firing rates (PreLesion - PreL) and the subsequent post lesion sessions (PoL1–3 and PreS) in RFA. The central black dot indicates the median, the box limits indicate the 25th and 75th percentiles. Whiskers represent the 5th and the 95th percentiles. **D)** LvR calculated between baseline firing rates (PreLesion - PreL) and the subsequent post lesion sessions (PoL1–3 and PreS) in S1. In **E1**) top sketch for CTR group and in the bottom sketch in **E2)** for ADS, LvR distribution shown as histogram with a bin size of 0.05 and determined across all the single units belonging to RFA during pre and post stimulation phase. **E3)** Box plot for the CTR group and ADS of the LvR calculated between pre stim firing rates (PreS) and immediately post stim session (PoS) in RFA. The central black dot indicates the median, the box limits indicate the 25th and 75th percentiles. Whiskers represent the 5th and the 95th percentiles. In **F1)** For CTR and in **F2)** for ADS, LvR distribution shown as histogram with a bin size of 0.05 and determined across all the single units belonging to S1 during pre and post stimulation phase. **F3)** For both CTR and ADS, LvR calculated between pre stim firing rates (PreS) and immediately post stim session (PoS) in S1. Friedman test for panels C and D: not significant; *p < 2.5·10− 2, **p < 5·10− 3 Wilcoxon rank-sum test for panels **E3** and **F3**

To further investigate possible changes in the firing patterns induced by either the lesion or the stimulation, we classified the type of spike-firing pattern of recorded neurons in both RFA and S1 through the LvR coefficient. As reported in Fig. 6, neurons of both RFA and S1 exhibited stable baseline firing patterns Pre Lesion, with values defining a “Random” state (cf. Figure 6A: LvR = 1.21 ± 0.25 and Fig. 6B: LvR = 1.12 ± 0.19, mean ± SD). After the lesion, the level of LvR, measured both within RFA and S1 was constant for the entire duration of the experiment (cf. Figure 6C, D). The effects of stimulation on LvR are reported in Fig. 6E and F. As demonstrated in the histograms of Fig. 6E1, CTR experiments showed an overlap of the distribution obtained during the PreS and PoS conditions. In the ADS experiments, there was a shift of the distributions towards the right indicating a global increase of LvR values in RFA, moving the patterns of activity from the “Random” condition of the PreS to the “Bursty” state of firing in PoS (Fig. 6E2). The quantification of this change was obtained by comparing the LvR PreS/PoS ratio between the CTR and ADS groups (Fig. 6E3). We found a statistically significant increase of ADS with respect to CTR (ADS vs CTR: **p < 5·10^− 3^ Wilcoxon rank-sum test). In S1 we obtained results similar to those obtained in RFA. The histograms show a strong overlap in the PreS and PoS distributions of LvR for CTR experiments while the distribution shifted towards higher LvR values in the ADS group (Fig. 6F1 and 6F2 respectively). By comparing the LvR PreS/PoS ratio between the CTR and ADS groups (Fig. 6F3), we found a statistical increase of ADS with respect to CTR (ADS vs CTR: **p < 5·10^− 3^ Wilcoxon rank-sum test).

## Discussion

The purpose of this study was to investigate alterations in single-unit firing properties of neurons within ipsilesional spared sensorimotor areas immediately following an ischemic injury to M1. During an ischemic event, neurons within the penumbra can display altered firing patterns, with a cessation of activity as blood flow is decreased (Heiss, Hayakawa et al. 1976). While the likelihood of neuronal survival in the ischemic core is dependent on reperfusion of blood flow, any significant alteration in activity will likely cause a subsequent disruption in the firing patterns of neurons that were connected to the affected region (Bauer, Kraft et al. 2014). Further, it is well established that ischemic injury can lead to an increase in glutamatergic release in the penumbra which can lead to an increase in activity, which can then feedback into an excitotoxic response in the hours immediately after injury (Rothman and Olney 1986). ET-1 injected into CFA induced a permanent ischemic injury, which led to rapid cell death in the core. Because CFA has strong, reciprocal projections to ipsilateral somatosensory and motor areas, the rapid loss of any inhibitory outputs to these areas, combined with any lesion-related glutamatergic response would suggest that an increase in the activity in both of these regions should be observed. Interestingly, RFA showed an increase of the global mean firing rate in the periods immediately after injury while S1 showed a consistent decrease over the same periods. There are a number of potential explanations for the variability in response. First, these areas are some distance from the ischemic core and are likely to have a more complex and variable response to an array directly in the penumbra. Second, the methodology for inducing the lesion across M1 takes a minimum of roughly 30 min to complete across the six injection locations. Therefore there is likely to be some timing differences between when more rostral or caudal areas of M1 are impacted, leading to potentially different results in PM and S1. Further analysis on individual units indicated that over half of the identified units increased their firing rate over the post-lesion period in both RFA and S1 even as the overall mean firing rate changed. This may be the result of neuronal sub-types having differential responses to the injury.

ADS is a potent way to synchronize the output of one neuron with the forced, evoked activity (through electrical stimulation) of another population of neurons. This was first demonstrated by (Jackson, Mavoori et al. 2006), to alter motor output within M1 of the macaque, and was further utilized by Guggenmos (2013), as a way to facilitate recovery after M1 injury in the rat by coupling premotor and somatosensory areas. While behavioral demonstrations offer the most direct indication of the efficacy of this approach, the underlying mechanisms, while based on spike-timing dependent plasticity, are still under investigation. We have previously described the impact of ADS on firing patterns both in anesthetized and ambulatory brain-intact rats. In the normal anesthetized case, ADS induces an increase in the overall MFR while decreasing the observed LvR in RFA, indicating that triggered stimulation in S1 was able to alter activity patterns in RFA even after stimulation ceased. Here, we observed a similar response in MFR in both RFA and S1 while LvR increased (or became more bursty) in RFA.

This trend in LvR to become more bursty is likely due to the observed shift in activity of the ADS (but not CTR) to induce a cyclic pattern of activity in the sub 1 Hz range. The majority of ADS animals showed this cyclic pattern of activity, but it is unclear what is underpinning these bursts. If it were merely a carry-over effect from the ADS, the stimulation frequency and subsequent entrainment should have been at higher (2-10 Hz) frequencies. (Carmichael and Chesselet 2002) described these low frequency waveforms with similar subsequent bursting of activity in peri-lesional cortex at one day post-injury with a thermocoagulatory lesion to CFA. These low-frequency waveforms are thought to synchronize neural activity that would ultimately shape neuroplastic mechanisms such as axonal sprouting. Our data suggests that ADS may be able to induce these patterns of synchronous activity rapidly after injury. Further investigation in both stimulated and non-stimulated animals is needed to assess the role stimulation may have in promoting and shaping these effects.

## Conclusion

In this study, we performed acute experiments in anesthetized rats affected by a focal lesion in the motor area. We recorded the electrophysiological activity of both premotor (RFA) and somatosensory (S1) cortex, in order to evaluate *(i)* how the focal lesion affects the activity of the two areas and *(ii)* whether acute closed-loop intracortical microstimulation (i.e. Activity-Dependent Stimulation, ADS) can alter the lesion induced changes. We first evaluated the immediate, global impact on neural activity of the focal lesion. We found that the ischemic injury resulted in an overall increase in the MFR within RFA but a decrease in S1, while simultaneously observing that the largest proportion of individual neurons increased their firing rates. Stimulation induced a further increase in the global MFR and the proportion of individual units increasing their firing. Stimulation also resulted in a shift in the pattern of activity from random to more bursty. Coupled with the observation of low-frequency synchronous activity within MEAs, it is clear that ADS can rapidly alter the intrinsic neural activity after a focal lesion. This work contributes to our growing understanding of how stimulation paradigms alter damaged brain networks and represents a fundamental step for the development of future therapies for acquired brain injury.

## Data Availability

Data collected and analyzed from this study are available on reasonable request.

## Abbreviations

M1: Primary Motor cortex
ADS: Activity-Dependent Stimulation
MEA: Microelectrode Array
RFA: Rostral Forelimb Area
S1: Somatosensory cortex
TBI: Traumatic Brain Injury
CFA: Caudal Forelimb Area
ET-1: Endothelin-1
CTR: Control group
CSF: Cerebrospinal Fluid
PTSD: Precise Timing Spike Detection
MFR: Mean Firing Rate
LVR: Local Variation compensate for Refractoriness.
